# Military Tract Medical Association

**Published:** 1866-08

**Authors:** 


### MILITARY TRACT MEDICAL ASSOCIATION.

The leading physicians of the counties of Bureau, Henry, Knox and Stark, Illinois, met at Kewanee, May 22d, 1866, and organized a district association, designated the Military Tract Medical Association. The following gentlemen were elected officers of the society:

*President*—A. H. Thompson, M. D., Princeton.

*Vice-President*—H. Nance, M. D., Kewanee.

*Secretary and Treasurer*—G. H. Scott, M. D., Kewanee.

*Censors*—N. Holton, M. D., Buda; Jno. M. Morse, M. D., Galesburg; V. C. Secord, M. D., Galva.

Adjourned to meet in Galesburg, on the second Tuesday in December.

